# Sintering and Mechanical Properties of (SiC + TiC_x_)_p_/Fe Composites Synthesized from Ti_3_AlC_2_, SiC, and Fe Powders

**DOI:** 10.3390/ma14092453

**Published:** 2021-05-09

**Authors:** Mingtao Wang, Zecheng Wang, Zhiyue Yang, Jianfeng Jin, Guoping Ling, Yaping Zong

**Affiliations:** 1 School of Materials Science and Engineering, Northeastern University, Shenyang 110819, China; WangZC18842511360@163.com (Z.W.); YZY950104@126.com (Z.Y.); jinjf@atm.neu.edu.cn (J.J.); zongyp@mail.neu.edu.cn (Y.Z.); 2 State Key Laboratory of Rolling and Automation, Northeastern University, Shenyang 110819, China; 3 Jiangyin Innovation Institute of Metal Materials Co., Ltd., Jiangyin 214400, China; Lingguoping_123@163.com

**Keywords:** iron matrix composite (IMC), reinforcing particles, MAX phase of Ti_3_AlC_2_, SiC, resistance sintering

## Abstract

Ceramic-particle-reinforced iron matrix composites (CPR-IMCs) have been used in many fields due to their excellent performance. In this study, using the fast resistance-sintering technology developed by our team, iron matrix composites (IMCs) reinforced by both SiC and TiC_x_ particles were fabricated via the addition of SiC and Ti_3_AlC_2_ particles, and the resulting relative densities of the sintering products were up to 98%. The XRD and EDS analyses confirmed the in situ formation of the TiC_x_ from the decomposition of Ti_3_AlC_2_ during sintering. A significant hybrid reinforcing effect was discovered in the (SiC + TiC_x_)_p_/Fe composites, where the experimental strength and hardness of the (SiC + TiC_x_)_p_/Fe composites were higher than the composites of monolithic SiC_p_/Fe and (TiC_x_)_p_/Fe. While, under the condition of constant particle content, the elongation of the samples reinforced using TiC_x_ was the best, those reinforced by SiC was the lowest, and those reinforced by (SiC + TiC_x_) fell in between, which means the plastic response of (SiC + TiC_x_)_p_/Fe composites obeyed the rule of mixture. The successful preparation of IMCs based on the hybrid reinforcement mechanism provides an idea for the optimization of IMCs.

## 1. Introduction

With its advantages of high strength and elasticity modulus, excellent abrasion performance, and great heat resistance, ceramic particle reinforced iron matrix composites (CPR-IMCs) have shown great application prospects in machinery, mining, metallurgy, and other fields, and thus have attracted extensive attention from researchers [1,2,3,4,5]. Iron matrix composites (IMCs) are generally popularly fabricated using the liquid-phase process or conventional powder metallurgy [2,6,7]. The normal powder metallurgy and even hot isostatic press sintering both take a long time (usually lasts several hours) and result in severe interface reactions between the reinforcing particles and the matrix, which could weaken the reinforcing effect caused by the ceramic particles and reduce the properties of the IMCs [7]. As a result, the application of IMCs has been limited.

Our team has developed a fast resistance-sintering technology by which IMCs can be synthesized under a certain external pressure in the condition of a semi-molten state. This sintering process, which is normally done in a few minutes, makes manufacturing feasible with low costs and produces excellent material properties due to the short processing time [5,8]. With a good load-bearing effect in composites, SiC is a very common reinforcing agent in many metal base materials, such as iron [5,9], aluminum [10,11,12,13], magnesium [14,15], nickel [16,17], and zinc [18]. Our previous research results showed that SiC has the best enhancement effect compared with the common candidate reinforcements, such as TiC, TiN, and Cr_3_C_2_ in IMCs [8]. Furthermore, IMCs reinforced using a low SiC content (≤10%) have been successfully prepared based on this technology [5,8,9,19,20]. Since the heat source of this technology is the joule heat generated by metal powders passing by an electric current, the electric conductivity of reinforcing particles has a very important effect on the sintering performance, and the electrical resistance of SiC particles is high (up to 5.3 × 10^−6^ Ω·m under 293 K) [8]. As a result, it is reasonable to find that there is a decrease in electrical conductivity and joule heat, which causes a decline in the material performance when the SiC content exceeds 10% [5]. Generally, the hardness and the wear resistance of composites can be dramatically improved by increasing the amount of reinforced particles [1]. Thus, it is extremely challenging and interesting to add another reinforcing particle on the basis of IMCs reinforced by SiC without reducing the electrical conductivity of the as-prepared powder to further optimize the performance of IMCs. As we know, the reports on the effects are too few and too inconsistent to draw any conclusions. Ti_3_AlC_2_, one kind of MAX phase materials (M is a transition metal, A is an element from groups IIIA to VIA, and X is C or N), has a very high electrical conductivity ((2.87–3.45) × 10^−7^ Ω·m under 293 K) [21,22,23,24,25,26]. Research has found that an exothermic reaction between Fe and Ti_3_AlC_2_ is predominant above 763.1 °C, and Ti_3_AlC_2_ totally disappears and transfers into TiC_x_ and Fe (Al) when the temperature is up to 1045 °C [24]. Furthermore, TiC_x_ is also a commonly used reinforcement agent in IMCs and has a good reinforcing effect for metal matrix composites (such as titanium [27], high entropy alloys [28,29], and iron [30]), as it has excellent hardness, a high melting temperature (3147 °C), wear resistance, and thermodynamic stability, according to previous work [8]. Meanwhile, it is predicted that there is better interface bonding between the matrix and TiC_x_ that is formed by the decomposition of Ti_3_AlC_2_. Recently, Ti_2_AlC, which is another kind of MAX phase with similar physical properties and crystal structure to Ti_3_AlC_2_, has been used as the precursor to trigger in situ TiC_x_ in Inconel718, where enhanced mechanical properties could be successfully obtained [25]. This is a very inspiring work, indicating the feasibility of the strengthening method using in situ TiC_x_ decomposed by Ti_3_AlC_2_.

According to the features of electrical conductivity and decomposition in an iron matrix at elevated temperatures, Ti_3_AlC_2_ was added into IMCs in this work. Meanwhile, SiC, which is one of the best reinforcing particles in IMCs, was also added to obtain IMCs with a higher particle content and better mechanical properties, such as tensile strength and hardness. The primary objective of this study was to investigate the feasibility of fabricating ICMs reinforced by both SiC and in situ TiC_x_ based on the decomposition of Ti_3_AlC_2_. Three combinations (SiC + Fe, Ti_3_AlC_2_ + Fe and SiC + Ti_3_AlC_2_ + Fe) were designed for comparison and the total particle contents (vol.%) were chosen to be 15% and 20% (the ratio was 1:1 in a two-particle-type mixture). The microstructure and properties of the IMCs were also examined to evaluate the sintered products and reveal the strengthening mechanism.

## 2. Materials and Methods

### 2.1. Materials and Samples

The starting materials for the IMCs preparation were powders of iron, α-SiC, and Ti_3_AlC_2_. The powders of iron and SiC used in the experiment were all commercial powder products (iron was produced by Beipiao Shenglong Power Metallurgy Co., Ltd., Chaoyang, China, and the SiC was from Shanghai Colloid Chemical Plant, Shanghai, China) with a ≥99.85% purity rating. Ti_3_AlC_2_ powder was synthesized using the fluctuation method and the synthesis details are described in [31,32]. The average particle sizes of the SiC and Ti_3_AlC_2_ powders were ~10 μm and ~5 μm, respectively. The iron powders were commercial deoxidized powder with an average size of 50 μm and a maximum C content of 0.015 wt.%. Meanwhile, zinc stearate was added as a caking agent and lubricant. The ratios of powders in different samples are as follows (Table 1).

Each mixture of powders was mixed for 15 h in a planetary high-energy ball mill and then compressed into a block with a size of 60 × 30 × 10 mm^3^. The grinding ball material was GCr15 and the mass ratio of the ball to the material was 5:1 with a speed of 300 r/min during the dry grinding process. The fast resistance-sintering technology, based on the equipment adapted from the traditional hot-pressing furnace [6,9,10,11,12], was adopted for the sintering of the cold-pressed samples. The main parameters of the sintering process were pressure, voltage, heating, and holding time. An external pressure limited to 40 MPa was applied to obtain a product with a high relative density according to the performance of the ceramic die. A two-stage sintering process, during which the first stage can heat the powders into a semi-molten state and the second stage can hold the state under applied stress for a higher relative density, was adopted in this research. The heating time was 70 s, the holding time was 180 s in total, and the voltage was 6.0 V. The current was conducted and broken alternately during the holding stage, where the on–off ratio was 1 s:1.2 s.

### 2.2. Structure Characterization and Mechanical Testing

The microstructure of the composites was observed by means of optical microscopy (OM, GX71, Olympus, Tokyo, Japan) and scanning electron microscopy (SEM, JSM-6510A, JEOL, Tokyo, Japan). The distribution of elements, such as Fe, Ti, Si, and Al, were analyzed using an energy-dispersive X-ray spectrometer (EDXS, INCA 25X-Max50, Oxford, UK) mounted on the SEM. Meanwhile, phase analysis of the as-mixed powders and sintered samples was performed via X-ray diffraction (XRD, Smartlab 9, Rigaku, Tokyo, Japan) observations with Cu Kα radiation at 40 kV and 40 mA. Tensile tests were performed via an electronic universal testing machine (AG-XPLUS1000KN, Shimadzu, Kyoto, Japan) at room temperature with a crosshead separation rate of 0.3 mm/min. The specimens were machined into a dumbbell shape with a length of 60 mm, the gauge section was 25 mm and Φ5 mm, and the clamping parts had a size of nearly 17.5 mm and Φ8 mm at each end. The relative density of the sintered samples was tested using the Archimedes method with a density determination kit (OHAUS, Parsippany, NJ, USA) and Vickers hardness was measured using a digital Vickers hardness tester (DVH, HVS-1000A, Huayin, Laizhou, China) with a load of 200 gf for 15 s. Each relative density was tested more than three times to ensure the repeatability and reliability of the data.

## 3. Results

### 3.1. Analysis of Mixed Powders

**Figure 1:**
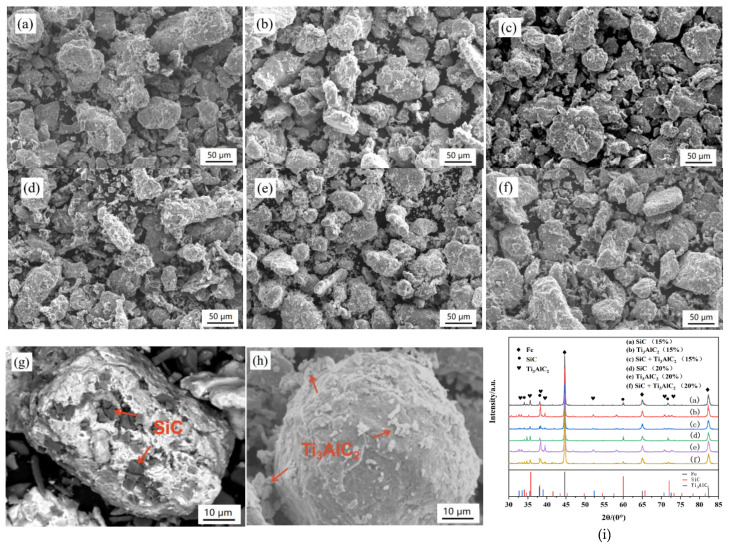
SEM micrographs and XRD patterns of mixed particles after ball-milling: (**a**) 15% SiC, (**b**) 15% Ti_3_AlC_2_, (**c**) 15% (SiC + Ti_3_AlC_2_), (**d**) 20% SiC, (**e**) 20% Ti_3_AlC_2_, (**f**) 20% (SiC + Ti_3_AlC_2_), (**g**) close-up of SiC and Fe powers, (**h**) close-up of the Ti_3_AlC_2_ and Fe powders, and (**i**) XRD results.

The mixed powders were ball-milled via the process introduced in Section 2.1 and the micro-morphologies of the mixed powders after ball-milling are shown in Figure 1. It can be seen that most of the iron powders were approximate ellipsoid and the reinforcements were uniformly distributed. No obvious particle agglomerating or iron powder breakage could be observed. According to Figure 1, no obvious size change happened during the ball milling. Most of the reinforcing agents were adsorbed onto the surface of the iron powder or embedded into them. Normally, SiC can be embedded in the iron powders because of its higher hardness and Ti_3_AlC_2_ can be adsorbed on the surface of the iron powders (see Figure 1g,h). Meanwhile, a few individual SiC and Ti_3_AlC_2_ particles were seen, which means that an appropriate ball milling process was chosen. Meanwhile, a phase analysis was carried out via XRD, where only Fe, SiC, and Ti_3_AlC_2_ could be found according to Figure 1i. Thus, we are sure that there was no new phase formed during the milling process.

### 3.2. Microstructure and Phase Components of the IMCs

**Figure 2:**
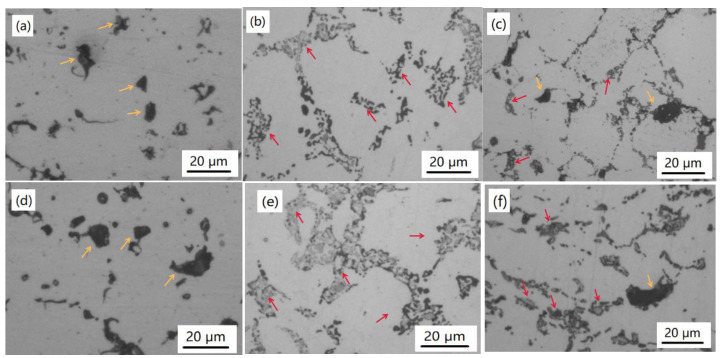
Microstructures of the IMCs supplemented with (**a**) 15% SiC, (**b**) 15% Ti_3_AlC_2_, (**c**) 15% (SiC + Ti_3_AlC_2_), (**d**) 20% SiC, (**e**) 20% Ti_3_AlC_2_, and (**f**) 20% (SiC + Ti_3_AlC_2_). SiC is indicated by the yellow arrow and TiC_x_ is indicated by the red arrow.

The distribution of the reinforcing particles in the iron matrix was observed using OM, where the microstructure is shown in Figure 2. The density of the reinforcing particles was similar under the same particle content and more reinforcing particles appeared in the sample with a higher particle content, i.e., the density in Figure 1a–c was 14.3–15.4% (area percentage) and the corresponding value in Figure 1d–f is 18.3–19.2%. In the samples, SiC exited in the form of granular particles with a size of ~10 μm (Figure 2a,d) and was dispersed randomly throughout the matrix, which is consistent with adding particles. There were distinct boundaries between the matrix and the SiC, which means a weak interface reaction [5,9]. Irregular-shaped islands could be seen in the samples with Ti_3_AlC_2_ (marked by red arrows in Figure 2b,c,e,f), which could have been caused by the decomposition of Ti_3_AlC_2_ (confirmed later). In addition, some decomposing products with a chain shape were also found in Figure 2b,c,e,f; the reason for this could be that the decomposing products were smaller and hence filled the gaps between iron powders easier under a certain pressure in the sintering process. In Figure 2c,f, both granular SiC and products decomposed by Ti_3_AlC_2_ can be observed.

**Figure 3:**
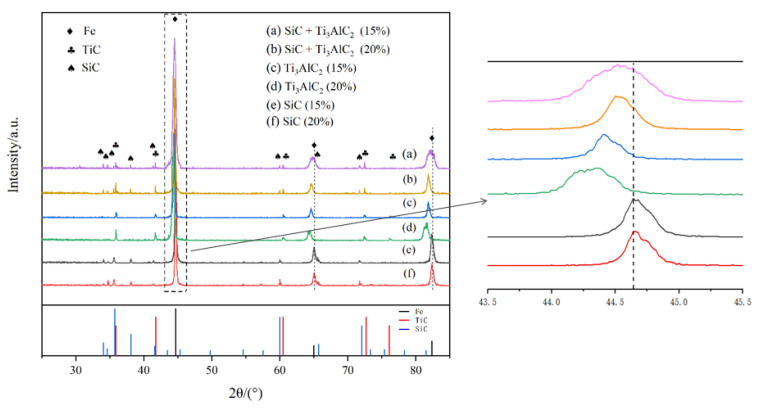
XRD patterns of the as-prepared samples supplemented with (**a**) 15% (SiC + Ti_3_AlC_2_), (**b**) 20% (SiC + Ti_3_AlC_2_), (**c**) 15% Ti_3_AlC_2_, (**d**) 20% Ti_3_AlC_2_, (**e**) 15% SiC, and (**f**) 20% SiC.

Comparatively speaking, it could be speculated that SiC and Ti_3_AlC_2_ presented different behaviors in the sintering process. SiC remained in the microstructure after sintering, while Ti_3_AlC_2_ could decompose according to the morphological features. In order to verify the behavior of the SiC and Ti_3_AlC_2_ during the sintering, an XRD test was carried out. According to the XRD analysis results in Figure 3, no trace of Ti_3_AlC_2_ could be detected in the samples with Ti_3_AlC_2_, indicating the complete decomposition of Ti_3_AlC_2_ in this sintering condition. Compared with the XRD results in Figure 1, it can be known that no new phase appeared during both the ball-milling and sintering in the SiC case; however, Ti_3_AlC_2_ decomposed during the sintering process, although there was no new phase formed during the ball-milling. SiC and TiC_x_ were the main phases in the sintered samples, which means that SiC was stable in the iron matrix and Ti_3_AlC_2_ decomposed to TiC_x_ during the sintering process. Because the solubility limit of Al in Fe is nearly 20 at.%, no new phase between Fe and Al was detected, and this conclusion was true for all the samples prepared in our research. According to the enlarged range of 43–45° (2θ) in Figure 3, the 2θ locations of diffraction peaks for the iron matrix shifted to lower 2θ angles in the samples with Ti_3_AlC_2_. According to the Bragg formula, the 2θ displacement shifting to lower Bragg angles indicates an increase in distance between adjacent lattice planes, which can be ascribed to the distortion of the lattice due to the incorporation of entire Al atoms derived from Ti_3_AlC_2_ phases into the iron matrix lattice. The results agree well with previous studies showing that Al diffuses out from Ti_3_AlC_2_ to form an Fe (Al) solid solution and TiC_x_, with a theoretical composition of TiC_0.625_, which appears when the temperature exceeds 763.1 °C [24].

**Figure 4:**
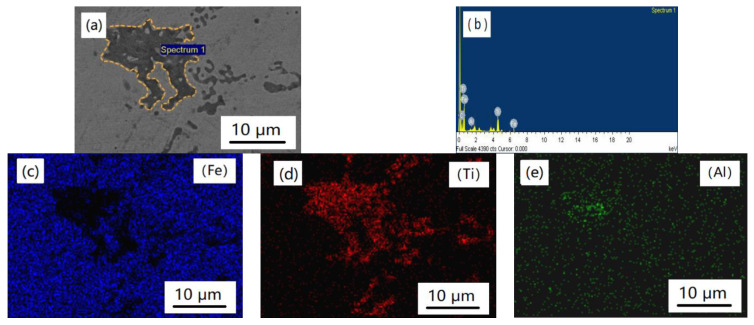
Morphology and EDS analysis results of TiC_x_ in the (Ti_3_AlC_2_ + SiC)/Fe composites: (**a**) micrograph, (**b**) EDS results of spectrum 1, (**c**) Fe, (**d**) Ti, and (**e**) Al.

**Figure 5:**
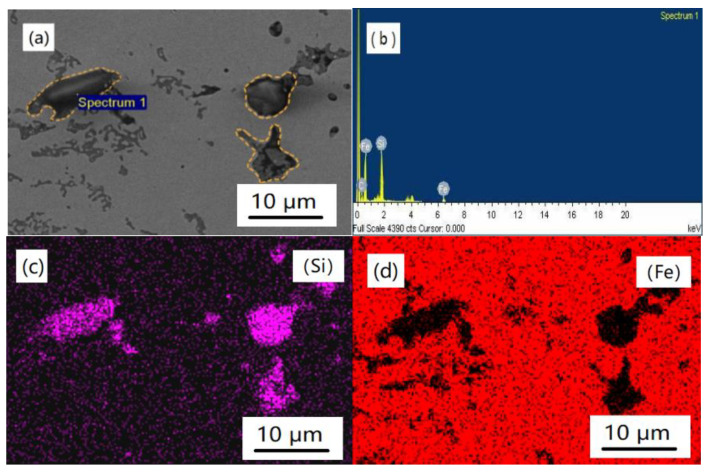
Morphology and EDS analysis results of SiC in the (Ti_3_AlC_2_ + SiC)/Fe composites: (**a**) micrograph, (**b**) EDS results of spectrum 1, (**c**) Si, and (**d**) Fe.

In order to further explore the reactions between the reinforcing particles and the iron matrix, an EDS analysis was carried out in the (Ti_3_AlC_2_ + SiC)/Fe composites. According to the EDS results in Figure 4 and Figure 5, it can be known that SiC was found, which agreed well with the OM observations and XRD results in Figure 2 and Figure 3. There was no reaction between the iron matrix and SiC, and similar results were also found in other kinds of metal-based materials reinforced by SiC [12,13,14,15,16], which means SiC is a stable reinforcer. As to Figure 5b,d, it shows that Ti was detected in the reinforcing particle area; however, Al was found in the whole observation area. It is clear that Ti_3_AlC_2_ decomposed according to the EDS map analysis results. This may be contributed to the diffusion relationship between Fe and Al, i.e., Al escaped from Ti_3_AlC_2_ and dissolved into the matrix and Fe near the particle could permeate into the Ti_3_AlC_2_ grains through the vacancy left by Al. A similar transformation was observed in a Cu–Ti_3_AlC_2_ system at elevated temperatures in which the Al de-intercalated from Ti_3_AlC_2_ along the (0001) basal plane to form a Cu (Al) solid solution [31]. Furthermore, in an Inconel718–Ti_2_AlC system, the Ti_2_AlC precursor completely transformed into an ultrafine TiC particulate via the dissolution of Al in the Ni matrix [25]. The decomposition reaction mechanisms of Ti_3_AlC_2_ in iron can be described as follows [24]:

Ti_3_AlC_2_ + Fe → TiC_0.625_ + Fe (Al) (1)

As a result, in situ TiC_x_ formed during the sintering process, which is consistent with the XRD analysis results and caused the morphological change of adding particles after sintering (Figure 2b,c,e,f).

**Figure 6:**
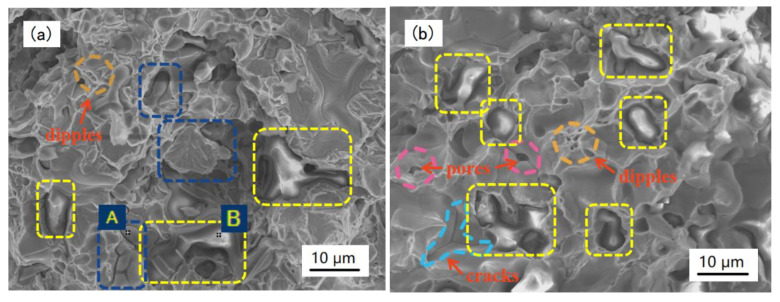

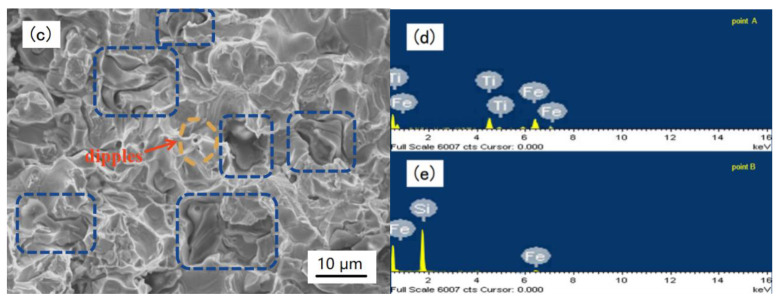
SEM images of the fracture surface of the samples with (**a**) 20% (Ti_3_AlC_2_ + SiC), (**b**) 20% SiC, (**c**) 20% Ti_3_AlC_2_, (**d**) EDS results of point A in (**a**), and (**e**) the EDS results of point B in (**a**). SiC is indicated by the yellow rectangle box and TiC_x_ is indicated by the blue rectangle box in (**a**–**c**).

After the tensile test, an analysis of the characteristics of the particles on the fracture surfaces was carried out. Typical microstructures with a 20% particle content can be seen in Figure 6, which illustrates the principally brittle morphology in all the samples, although small dimples appeared on the fracture surfaces due to the iron matrix deformation features. Figure 6a shows that both the SiC and TiC_x_ particles were dispersed on the fracture surface. According to the surface morphology and EDS analysis in Figure 6a,d,e, many TiC_x_ particles were broken and most SiC particles were unbroken. During the tensile process, a “tear ridge” was formed by the plastic deformation around the particles and the particles were embedded in the surface in the form of “breakage” (most are TiC_x_) or “pull-out” (most are SiC) modes. It can be deduced that both kinds of particles served as the main contributors as load carriers when the fracture occurred, which means the reinforcing effect was achieved by the particles via a load transfer mechanism.

It can be seen in Figure 6b that most of the SiC particles were unbroken and some ridges that formed via deformation around the particles can be seen, which means that the particles acted as a reinforcement, mainly by means of the “pull-out” mode. It can be concluded that decohesion occurred at the interfaces when the fracture strength in the particles exceeded the interfacial strength. Meanwhile, some small pores and cracks whose surrounding matrix did not deform were also observed, as shown in Figure 6b. This could have been caused by inadequate sintering. In Figure 6c, many TiC_x_ particles are shown to be broken, which means the particles bore the load during the deformation. It is reported that the fracture mode depends on the relative strength of the interface and the reinforcing particles [30] in composites. The fracture of the particles occurred in this work, suggesting that the interfacial strength in the new composites was so high that it exceeded the threshold stress of TiC_x_ particles for fracture. Thus, it can be concluded that the in situ TiC_x_ composites had strong interfacial bonding forces, which resulted in a cleavage fracture of in situ TiC_x_ particles rather than a “pull-out” fracture at the interfaces between the in situ TiC_x_ particles and the iron matrix.

According to Figure 6a–c, the difference in the particle morphology could be attributed to the strength of the particle and its combination with the iron matrix. A weaker strength and firm bond would cause a breakage, such as in the case of the TiC_x_ particles. As to the SiC particles, a “pull-out” effect means a higher strength and weaker bond with the matrix.

### 3.3. Properties of the IMCs

**Figure 7:**
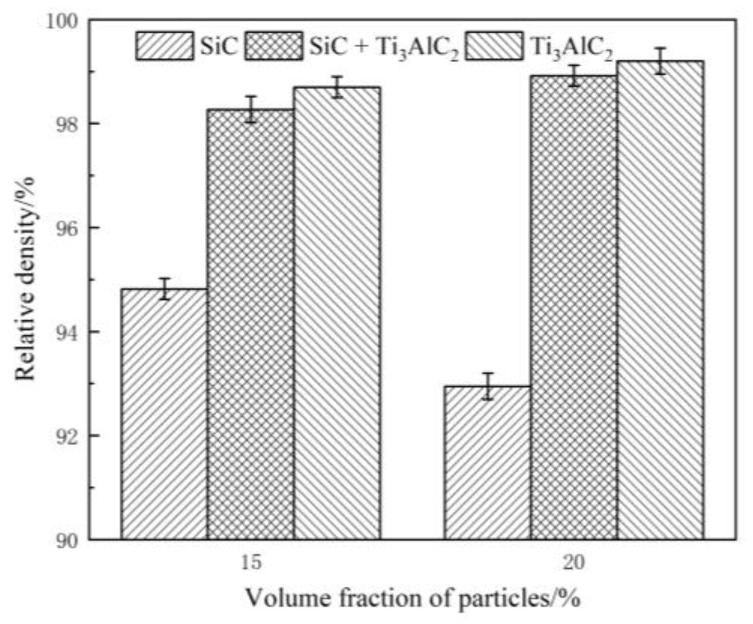
The relative densities of the as-prepared samples.

Figure 7 shows the relative density of samples with different particle types and contents. It can be seen that the relative density decreased with an increasing particle content in the samples that were reinforced only by SiC (from 95 to 93%). In the cases of samples that were created by adding Ti_3_AlC_2_, the relative densities were high and up to 98%. The reason was that the heat mainly came from the joule heat produced by the current through the iron powders. With the increasing content of reinforcing particles, the electrical resistance of the samples that were reinforced only by SiC decreased and the heating effect became weak. In contrast, Ti_3_AlC_2_ has a lower electrical resistance and thus a better sintering effect can be achieved. Meanwhile, the worse sintering effect means more sintering defects, such as pores, which can be seen in Figure 6b [5].

**Figure 8:**
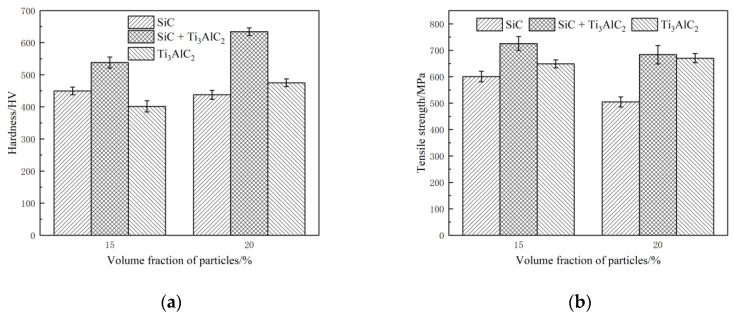
The hardness and tensile strengths of the as-prepared samples. (**a**) hardness; (**b**) tensile strength.

Then, the hardness and strength, as important properties of wear-resisting materials, were tested and the results are shown in Figure 8. For the same volume content, samples reinforced by both SiC and TiC_x_ had the highest hardness and strength, which were 550 HV and 720 MPa and 635 HV and 683 MPa on average, respectively. Both of these properties exhibit a hybrid reinforcement effect, i.e., samples reinforced by SiC and TiC_x_ were better than samples with a single reinforcing agent. As to the effect of particle content, the IMCs with different reinforcing agents exhibited varied performance trends in our research. In the case of samples reinforced by SiC, the hardnesses and strengths were 450 HV and 600 MPa and 438 HV and 500 MPa on average, respectively, which showed a decrease with increasing content. For the samples reinforced by TiC_x_, the properties increased with a content increase, i.e., their values were 400 HV and 649 MPa and 475 HV and 670 MPa on average, respectively. With regard to the samples reinforced by both SiC and TiC_x_, the hardness increased and the strength decreased with increasing content.

This interesting phenomenon was mainly determined by the characteristics of the fast resistance-sintering technology. During the sintering process, there are two critical factors contributing to the mechanical performance: the types of reinforcing particles and electrical conductivity. On the one hand, the improvement in hardness and strength requires stronger particles with a better load-bearing ability and higher content [8]. A load can be transferred from the matrix to the SiC and TiC_x_ reinforcements, which is expected to produce high strength and hardness in the composites [11]. On the other hand, high electrical conductivity is also needed to generate sufficient joule heat for the sintering effect. For the case of adding only SiC, the joule heat will decrease with an increasing SiC content because of its high electrical resistance. Based on our previous results, the hardness and strength of the IMCs reinforced by SiC first increased and then decreased with an increasing SiC content, and the optimal content was 10% [5]. The electric resistance of Ti_3_AlC_2_ was much lower than SiC, which meant that effective sintering could be obtained. Furthermore, an exothermic reaction was predominant in the process when the temperature was above 763.1 °C [24], which could also provide the energy required for sintering. Thus, the hardness and strength of samples supplemented with Ti_3_AlC_2_ increased with increasing content when the content was 15–20%, as was the case in this research.

TiC_x_ leads to an inferior strengthening effect compared to SiC, as confirmed by the experimental data when the content is not more than 10%. When the content was 15–20% as was the case in this research, the strength and hardness of the samples supplemented with Ti_3_AlC_2_ were higher than with SiC, which means that the particle reinforcement was determined by the combined effect of both the particle strength and electrical resistance, as mentioned above. Therefore, the addition of SiC ensured the strength of the IMCs. The addition of Ti_3_AlC_2_ ensured that there was enough joule heat caused by the electrical current and strengthening of both particles and the solid solution. As a result, a hybrid strengthening feature caused by TiC_x_ and SiC could be achieved using this sintering technology. Moreover, the strengthening mechanism based on the decomposition of Ti_3_AlC_2_ has multiple effects. According to the first-principles calculation of sub-stoichiometric TiC_x_ [33], the bulk modulus of relaxed TiC_0.625_ is 226 GPa, which is much higher than that of Ti_3_AlC_2_ (160 GPa). After the decomposition, there was a large amount of bulky TiC_x_, as seen in Figure 6, which could play the role of transferring the load in the composites. Meanwhile, the strength of the Fe (Al) solid solution could be better than the Fe matrix due to solution strengthening. In addition, the interfacial layer formed in this way provided a high-strength transient zone between the Fe matrix and TiC_x_, favoring the load transfer in the composite during deformation. As a result, a better interface bonding between the in situ TiC_x_ and the matrix was expected because of the formation mechanism of TiC_x_ discussed above, which could benefit the tensile strength and hardness.

**Figure 9:**
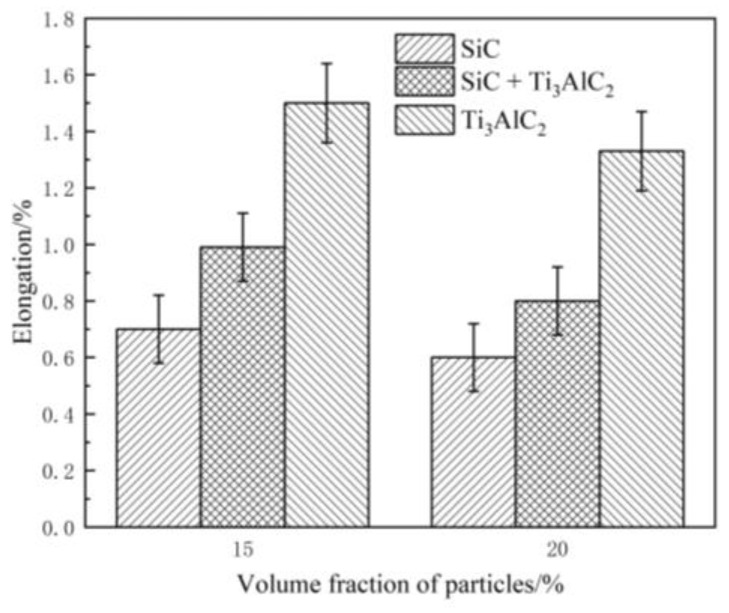
The elongation of the as-prepared samples.

According to Figure 9, the elongation of samples that were supplemented with Ti_3_AlC_2_ was the highest, followed by samples with SiC + Ti_3_AlC_2_ and the sample with SiC was the lowest with the same content, which means the elongation followed the rule of mixture. When reinforced by the same particle type, the plasticity of the IMCs became worse with an increase in the content. As a result, the plasticity of the IMCs also mainly relied on the particle content and electrical resistance, where a high content meant more interfaces between the particles and the matrix, inevitable agglomeration, and more sintering defects, such as pores and cracks. With a certain content, different particles meant varied electrical resistances of the samples, where a lower powder electrical resistance would produce a better sintering effect with fewer defects.

To sum up, we aimed to increase the content of reinforcing particles in IMCs prepared using the fast resistance-sintering technology in this study. Ti_3_AlC_2_, with excellent electrical conductivity and a feature of decomposition into TiC_x_ at elevated temperatures, was chosen, together with SiC, to be added into an iron matrix. The present approach provides an effective route for fabricating IMCs that are reinforced by SiC and in situ TiC_x_ particulates. The experimental results show that the addition of SiC and Ti_3_AlC_2_ could achieve the hybrid strengthening effect and the IMCs products reinforced by SiC and TiC_x_, formed by the decomposition of Ti_3_AlC_2_, could be obtained. The successful preparation of IMCs based on the hybrid reinforcement mechanism provides an idea for the optimization of IMCs.

## 4. Conclusions

1. IMCs reinforced by both SiC and TiC_x_ were prepared successfully through the addition of SiC and Ti_3_AlC_2_ particles with volume fractions of 15% and 20% via the fast resistance-sintering technology, where the relative density of the samples was up to 98%.
2. The in situ formation of the TiC_x_ from the decomposition of Ti_3_AlC_2_ during sintering was confirmed based on the XRD and EDS analysis results.
3. The hybrid reinforcing effect, i.e., the tensile strength and hardness of samples reinforced by the mixture of SiC and TiC_x_ were better than using a single reinforcing agent with the same volume content, was achieved. The elongation of the samples reinforced by TiC_x_ was the best, followed by SiC and TiC_x_, and those reinforced by SiC were the lowest, which means the elongation obeyed the rule of mixture.

## Figures and Tables

**Table 1:** The ratios of powders in the different samples (vol.%).

Particles	15%	20%	15%	20%	15%	20%
	SiC	SiC	Ti_3_AlC_2_	Ti_3_AlC_2_	(SiC + Ti_3_AlC_2_)	(SiC + Ti_3_AlC_2_)
SiC	15%	20%	-	-	7.5%	10%
Ti_3_AlC_2_	-	-	15%	20%	7.5%	10%
Fe	Balance					

## Data Availability

Not applicable.

## References

[1] Zheng Y., Zhou Y., Li R., Wang J., Chen L., Li S. (2017). Preparation and Mechanical Properties of TiC-Fe Cermets and TiC-Fe/Fe Bilayer Composites. J. Mater. Eng. Perform..

[2] Das K., Bandyopadhyay T.K., Das S. (2002). A Review on the various synthesis routes of TiC reinforced ferrous based composites. J. Mater. Sci..

[3] Oh N.R., Lee S.K., Hwang K.C., Hong H.U. (2016). Characterization of microstructure and tensile fracture behavior in a novel infiltrated TiC-steel composite. J. Scr. Mater..

[4] Prabhu T.R., Varma V.K., Vedantam S. (2014). High-Speed Tribological and Mechanical Properties of Layered Fe/SiC Composites. J. Mater. Eng. Perform..

[5] Wang Y.M., Zong B.Y., Zhuang Y.B., Li J. (2011). Effects of content and size of reinforcing particles on mechanical properties of SiCp_Fe composites processed by dynamic hot press sintering. J. Mater. Sci. Technol..

[6] Talas S. (2006). Microstructural characterization of SiC added structural steel. Kovove Mater..

[7] Patankar S.N., Tan M.J. (2000). Role of reinforcement in sintering of SiC/316L stainless steel composite. Powder Met..

[8] Li J., Zong B.Y., Wang Y.M., Zhuang W.B. (2010). Experiment and modeling of mechanical properties on iron matrix composites reinforced by different types of ceramic particles. Mater. Sci. Eng. Struct..

[9] Zhuang W.B., Zong B.Y., Wang Y.M., Yang Y.F. (2013). Processing and properties of SiC_p_/Fe composites by resistance sintering with a novel dynamic temperature control. J. Compos. Mater..

[10] Sangmin S., Hyeonjae P., Byeongjin P., SangBok L., SangKwan L., Yangdo K., Seungchan C., Ilguk J. (2021). Dispersion Mechanism and Mechanical Properties of SiC Reinforcement in Aluminum Matrix Composite through Stir- and Die-Casting Processes. J. Appl. Sci..

[11] Yao X., Zhang Z., Zheng Y.F., Kong C., Quadir M.Z., Liang J.M., Chen Y.H., Munroe P., Zhang D.L. (2017). Effects of SiC Nanoparticle Content on the Microstructure and Tensile Mechanical Properties of Ultrafine Grained AA6063-SiC_np_ Nanocomposites Fabricated by Powder Metallurgy. J. Mater. Sci. Technol..

[12] Huang C.W., Aoh J.N. (2018). Friction Stir Processing of Copper-Coated SiC Particulate-Reinforced Aluminum Matrix Composite. Materials.

[13] Winter L., Hockauf K., Lampke T. (2018). Temperature and Particle Size Influence on the High Cycle Fatigue Behavior of the SiC Reinforced 2124 Aluminum Alloy. Metals.

[14] Kamrani S., Hubler D., Ghasemi A., Fleck C. (2019). Enhanced Strength and Ductility in Magnesium Matrix Composites Reinforced by a High Volume Fraction of Nano- and Submicron-Sized SiC Particles Produced by Mechanical Milling and Hot Extrusion. Materials.

[15] Subramani V., Jayavel B., Sengottuvelu R., Lazar P.J.L. (2019). Assessment of Microstructure and Mechanical Properties of Stir Zone Seam of Friction Stir Welded Magnesium AZ31B through Nano-SiC. Materials.

[16] Zhang H.Q., Niu X.X., Pei Z.L., Shi N.L., Gong J., Sun C. (2020). Effects of Cr and Al Contents on the Preparation of SiC Fiber-Reinforced NiCrAl Alloy Matrix Composite. Acta Met. Sin. Eng..

[17] Yang C., Huang H.F., De Los Reyes M., Yan L., Zhou X.T., Xia T., Zhang D.L. (2015). Microstructures and Tensile Properties of Ultrafine-Grained Ni-(1-3.5) wt % SiCN_p_ Composites Prepared by a Powder Metallurgy Route. Acta Met. Sin. Eng..

[18] Gao C.D., Yao M., Shuai C.J., Peng S.P., Deng Y.W. (2019). Nano-SiC reinforced Zn biocomposites prepared via laser melting: Microstructure, mechanical properties and biodegradability. J. Mater. Sci. Technol..

[19] Zhang Y., Zong B.Y., Jin J., Cao X. (2015). Electroless copper plating on particulate reinforcements and effects on mechanical properties of SiCp/Fe composite. Surf. Eng..

[20] Cao X.J., Jin J.F., Zhang Y.B., Zong B.Y. (2015). Mechanical properties of iron matrix composites reinforced by copper-coated hybrid ceramic particles. J. Mater. Res..

[21] Huang X.C., Feng Y., Qian G., Zhao H., Zhang J.C., Zhang X.B. (2018). Physical, mechanical, and ablation properties of Cu-Ti_3_AlC_2_ composites with various Ti_3_Al_C2_ contents. Mater. Sci. Technol..

[22] Radovic M., Barsoum M.W. (2013). MAX phases: Bridging the gap between metals and ceramics. Am. Ceram. Soc. Bull.

[23] Eklund P., Beckers M., Jansson U., Hogberg H., Hultman L. (2010). The M_(n+1)_AX_(n)_ phases: Materials science and thin-film processing. Thin. Solid Films.

[24] Chen X.H., Zhai H.X., Song P.F., Huang Z.Y. (2011). Reaction Behavior of Ti_3_AlC_2_ with Fe at High Temperature. Rare Met. Mater. Eng..

[25] Hu W.Q., Huang Z.Y., Yu Q., Wang Y.B., Jiao Y.D., Lei C., Cai L.P., Zhai H.X., Zhou Y. (2020). Ti_2_AlC triggered in-situ ultrafine TiC/Inconel 718 composites: Microstructure and enhanced properties. J. Mater. Sci. Technol..

[26] Wang H., Han H., Yin G., Wang C.Y., Hou Y.Y., Tang J., Dai J.X., Ren C.L., Zhang W., Huai P. (2017). First-Principles Study of Vacancies in Ti_(3)_SiC_(2)_ and T_i(3)_AlC_(2)_. Materials.

[27] Sun X.L., Han Y.F., Cao S.C., Qiu P.K., Lu W.J. (2017). Rapid in-situ reaction synthesis of novel TiC and carbon nanotubes reinforced titanium matrix composites. J. Mater. Sci. Technol..

[28] Wu H., Huang S.R., Zhu C.Y., Zhang J.F., Zhu H.G., Xie Z.H. (2020). In Situ TiC/FeCrNiCu High-Entropy Alloy Matrix Composites: Reaction Mechanism, Microstructure and Mechanical Properties. Acta Met. Sin. Eng..

[29] Zhang J.F., Jia T., Qiu H., Zhu H.G., Xie Z.H. (2020). Effect of cooling rate upon the microstructure and mechanical properties of in-situ TiC reinforced high entropy alloy CoCrFeNi. J. Mater. Sci. Technol..

[30] Lee J., Lee D., Song M.H., Rhee W., Ryu H.J., Hong S.H. (2018). In-situ synthesis of TiC/Fe alloy composites with high strength and hardness by reactive sintering. J. Mater. Sci. Technol..

[31] Wang X.H., Zhou Y.C. (2002). Solid-liquid reaction synthesis of layered machinable Ti3AlC2 ceramic. J. Mater. Chem..

[32] Zhang J., Wang J.Y., Zhou Y.C. (2007). Structure stability of Ti_3_AlC_2_ in Cu and microstructure evolution of Cu-Ti_3_AlC_2_ composites. Acta Mater..

[33] Hugosson H.W., Korzhavyi P., Jansson U., Johansson B., Eriksson O. (2001). Phase stabilities and structural relaxations in substoichiometric TiC1-x. Phys. Rev. B.

